# Plant sphingolipids promote extracellular vesicle release and alleviate amyloid-β pathologies in a mouse model of Alzheimer’s disease

**DOI:** 10.1038/s41598-019-53394-w

**Published:** 2019-11-14

**Authors:** Kohei Yuyama, Kaori Takahashi, Seigo Usuki, Daisuke Mikami, Hui Sun, Hisatoshi Hanamatsu, Junichi Furukawa, Katsuyuki Mukai, Yasuyuki Igarashi

**Affiliations:** 1 Lipid Biofunction Section, Faculty of Advanced Life Science, Hokkaido University, Kita-21, Nishi-11, Kita-ku, Sapporo 001-0021 Japan; 2 R & D Headquarters, Daicel Corporation, 2-18-1, Konan, Minato-ku, Tokyo 108-8230 Japan; 3 Department of Advanced Clinical Glycobiology, Faculty of Medicine, Graduate School of Medicine, Hokkaido University, Kita-21, Nishi-11, Kita-ku, Sapporo 001-0021 Japan

**Keywords:** Alzheimer's disease, Drug development

## Abstract

The accumulation of amyloid-β protein (Aβ) in brain is linked to the early pathogenesis of Alzheimer’s disease (AD). We previously reported that neuron-derived exosomes promote Aβ clearance in the brains of amyloid precursor protein transgenic mice and that exosome production is modulated by ceramide metabolism. Here, we demonstrate that plant ceramides derived from *Amorphophallus konjac*, as well as animal-derived ceramides, enhanced production of extracellular vesicles (EVs) in neuronal cultures. Oral administration of plant glucosylceramide (GlcCer) to APP overexpressing mice markedly reduced Aβ levels and plaque burdens and improved cognition in a Y-maze learning task. Moreover, there were substantial increases in the neuronal marker NCAM-1, L1CAM, and Aβ in EVs isolated from serum and brain tissues of the GlcCer-treated AD model mice. Our data showing that plant ceramides prevent Aβ accumulation by promoting EVs-dependent Aβ clearance *in vitro* and *in vivo* provide evidence for a protective role of plant ceramides in AD. Plant ceramides might thus be used as functional food materials to ameliorate AD pathology.

## Introduction

Alzheimer’s disease (AD) is a common form of dementia with a pathology characterised by the progressive intracerebral accumulation of amyloid-β protein (Aβ). This accumulation results from impaired clearance of Aβ in the sporadic form of AD and from increased production due to genetic mutations of amyloid precursor protein (APP) or Aβ processing enzymes in the less-common familial form of AD^1^. The imbalanced metabolism of Aβ that results in its accumulation is linked to tau pathology, neuronal impairment and the eventual emergence of the clinical symptoms of AD^2^. The prolonged preclinical stages of AD, including the phase of Aβ accumulation, provide a critical opportunity for preventive interventions.

Small extracellular vesicles (40–100 nm in diameter) called exosomes are released from various types of cells that have recently emerged as a key player for the intercellular transport of molecules both in health and in disease conditions, including AD^3,4^. In neuronal cultures and sera from AD patients, EVs associate with amyloid precursor protein (APP) and its metabolites, including C-terminal fragments (CTFs), amyloid intracellular domain and Aβ^5–7^. Our previous studies showed that extracellular vesicles (EVs) released from cultured neurons associate with Aβ through their surface glycosphingolipids (GSLs) and are incorporated into microglia for degradation^8,9^. Furthermore, APP transgenic mice infused continuously with neuron-derived EVs had decreased Aβ levels and amyloid deposition in their brains, suggesting that EVs mediate Aβ clearance^9,10^. We also reported that production of EVs in neurons is modulated by the metabolism of the sphingolipid family of membrane lipids. The inhibition of neutral sphingomyelinase-2 reduces ceramide and prevents exosome release, whereas knockdown of sphingomyelin synthase 2 (SMS2) increases ceramide and promotes exosome release^8,11^.

One of the major sphingolipids that is digested daily by humans is plant glucosylceramide (GlcCer)^12^. GlcCer, a type of GSL, has a single glucose attached to ceramide, which is hydrolysed into its components, glucose, a fatty acid and a sphingoid base, by digestive intestinal enzymes for uptake by intestinal enterocytes^13^. A portion of the sphingoid bases, including those of plant origins, is then resynthesized to ceramide, GlcCer and other complex sphingolipids such as sphingomyelin^14,15^. Sphingoid bases have diverse structures^16^. The most common sphingoid base in mammalian cells is sphingosine (trans-4-sphingenine, d18:1^4^), whereas plant sphingolipids consist of Δ8-unsaturated sphingoid bases such as 4,8-sphingadienine (d18:2^4,8^)^17,18^. Recent reports indicate that dietary plant sphingolipids have beneficial effects, providing improvements to the skin barrier and anticancer activity^19–22^.

In this study, we examined the effect of plant ceramides on EVs-dependent Aβ clearance to prevent AD pathogenesis. We showed that oral administration of plant GlcCer to APP transgenic mice markedly reduced Aβ levels and amyloid plaques and eventually attenuated Aβ-related pathologies, such as inflammation, synaptic dysfunction and cognitive deficits. We also demonstrated that plant sphingolipids increase the production of neuron-derived EVs with the ability to clear Aβ in neuronal cultures and in mice.

## Results

### Exogenously added plant ceramides increased release of EVs from neuronal cultures

**Figure 1:**
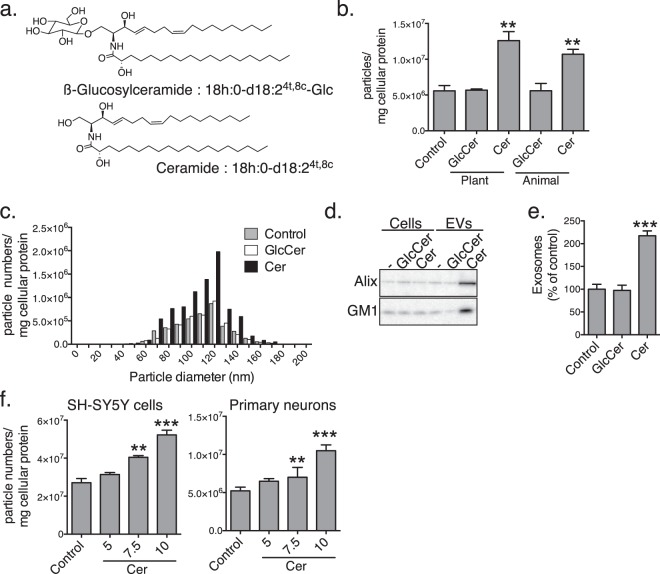
Plant ceramides induced release of neuron-derived EVs. (**a**) Chemical structures of typical GlcCer extracted from *Amorphophallus konjac* and its deviated ceramide. (**b**) Particle numbers of the EVs isolated from supernatants of SH-SY5Y cells treated for 24 h with GlcCer or ceramides. (**c**) Particle size distribution of the control and GlcCer- and ceramide-treated fractions of EVs. (**d**) Western blotting for Alix, CD63 and ganglioside GM1 exosome markers in supernatants from SH-SY5Y cells (1 × 10^5^/lane) and isolated EVs (from 1 × 10^7^ cells/lane). (**e**) Exosome amounts in culture supernatants of GlcCer- or ceramide-treated SH-SY5Y cells measured by PS-capture exosome ELISA system. (**f**) Particle numbers of the EVs isolated from supernatants of SH-SY5Y cells or primary neurons treated with the indicated concentrations of ceramides for 24 h. Data are presented as means ± SDs. **P < 0.01; ***P < 0.001 by *t* tests.

To examine the effects of plant ceramides on production of EVs, we treated human neuroblastoma SH-SY5Y cells with *Amorphophallus konjac*-derived GlcCer and ceramides at a concentration of 10 µM for 24 h. The ceramides were prepared from the GlcCer by glucose hydrolysis with EGCases (Fig. 1a)^18^. We previously showed that the sphingoid bases of konjac-derived GlcCer and Cer from these are composed of a major 4,8-sphingadienine (d18:2^4,8^) and, an minor 4-hydroxy-8-sphingenine (t18:1^8^), with combinations of C_16:0_, C_18:0_ and C_20:0_ fatty acids (Fig. 1a)^18^. Particle counting with a nanoparticle analyser revealed that treatment with the plant ceramides increased the amounts of EVs and animal-type ceramides (d18:1/C_18:0_) in supernatants from the cultured cells (Fig. 1b), whereas treatment with GlcCer, either plant or animal, had no effect. The particle sizes, ranging from 60 to 160 nm in diameter, with a peak at around 120 nm, remained similar between control and ceramide treatments (Fig. 1c). Western blot analysis showed that the levels of the exosomal markers Alix and ganglioside GM1 increased in the plant ceramide-treated EVs (Fig. 1d, Supplementary Fig. S1). In accordance, there was an approximately 2-fold increase in the number of EVs induced by the plant ceramides as determined by an exosome enzyme-linked immunosorbent assay (ELISA) using Tim4, a receptor for exosomal lipid phosphatidylserine, and an antibody against exosomal marker protein CD63 (Fig. 1e)^23^ (Fig. 1e). Furthermore, treatment with the plant ceramides induced a dose-dependent increase in the number of EVs collected from culture supernatants of SH-SY5Y cells as well as of mouse primary neurons (Fig. 1f).

### Plant ceramides promote EVs-dependent Aβ clearance

**Figure 2:**
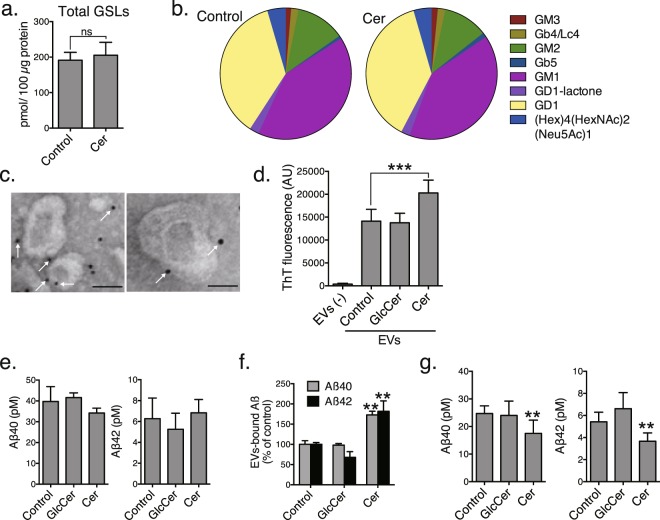
Plant ceramide-dependent release of EVs promotes Aβ clearance. Mass spectrometry analysis of total amounts of GSL-glycans (**a**) and relative GSL-glycan compositions (**b**) in EVs collected from control or ceramide-treated SH-SY5Y cell cultures. (**c**) Immunolabelling for Aβ on EVs derived from ceramide-treated cell cultures. Representative electron microscopic images are shown. Scale bar, 100 nm. (**d**) Thioflavin T (ThT) fluorescence to quantify amyloid fibrils in EVs collected from SH-SY5Y cell cultures (1 × 10^7^ cells) incubated with soluble Aβ and incubated for 24 h. ELISA measurements of total (**e**) and EVs-associated (**f**) Aβ levels in media from control and treated APP-expressing SH-SY5Y cells. (**g**) Aβ levels in medium from transwell cultured APP-expressing SH-SY5Y and BV-2 cells. Values are the means ± SEMs. **P < 0.01; ***P < 0.001 by *t* tests.

As our previous study demonstrated that surface GSLs are responsible for the association between EVs and Aβ^8^, we performed quantitative GSL glycomics to analyse the profiles of GSL-derived glycans from the EVs collected from control and ceramide-treated SH-SY5Y cells. The amount of total GSLs in the EVs did not change with plant ceramide treatment (Fig. 2a). Additionally, the GSL compositions in the EVs were quite similar between the control and the ceramide-treated conditions, indicating that plant ceramides increase the numbers of EVs without affecting their GSL profiles (Fig. 2b). Among the 8 GSL species detected in the EVs, most were sialylated species, such as GM1, GD1 and GM3, which have Aβ-binding abilities^24^. Electron microscopy revealed Aβ-immunopositive signals on the surfaces of the EVs isolated from plant ceramide-treated cell cultures that had been incubated with soluble synthetic Aβ40 at room temperature for 10 min (Fig. 2c). The binding of Aβ to neuron-derived EVs leads to Aβ amyloidogenesis with continued incubation with Aβ^8^. We also measured the amounts of Aβ amyloid fibrils in the supernatants of cells (1 × 10^7^ cells) incubated with Aβ40 at 37 °C for 15 h. The EVs derived from ceramide-treated cells formed greater amounts of amyloid fibrils than those from control or GlcCer-treated cells (Fig. 2d), suggesting that the plant ceramides, but not GlcCer, induce production of EVs that are able to bind Aβ.

We also previously demonstrated that exosome-bound Aβ is taken up by microglia in a phosphatidylserine-dependent manner, transported through the endocytic pathway and degraded in lysosomes^8^. To determine whether the increase in EVs induced by plant ceramide treatment promotes Aβ clearance, we first confirmed that the ceramide treatment did not alter extracellular concentrations of Aβ40 and Aβ42 by using SH-SY5Y cells overexpressing APP (Fig. 2e). The levels of EVs-bound Aβ40 and Aβ42 isolated from these cells were much higher than from control or GlcCer-treated cells (Fig. 2f), which may reflect the plant ceramide-induced increase in EVs. Next, we used a transwell culture system to see if EVs and Aβ secreted from APP-overexpressing SH-SY5Y cells placed on inserts can interact with microglial BV-2 cells placed at the bottom of the wells. The Aß levels were measured by ELISA after treatment of the transwell culture system with ceramides or GlcCer for 24 h. The extracellular concentrations of Aβ40 and Aβ42 after the treatment with plant ceramides were much lower than in controls or with GlcCer treatment (Fig. 2g). These data suggest that exogenously added plant ceramides accelerate EVs-dependent clearance by microglia and decrease extracellular Aβ.

### Dietary plant GlcCer reduces Aβ pathology in brains of APP transgenic mice

**Figure 3:**
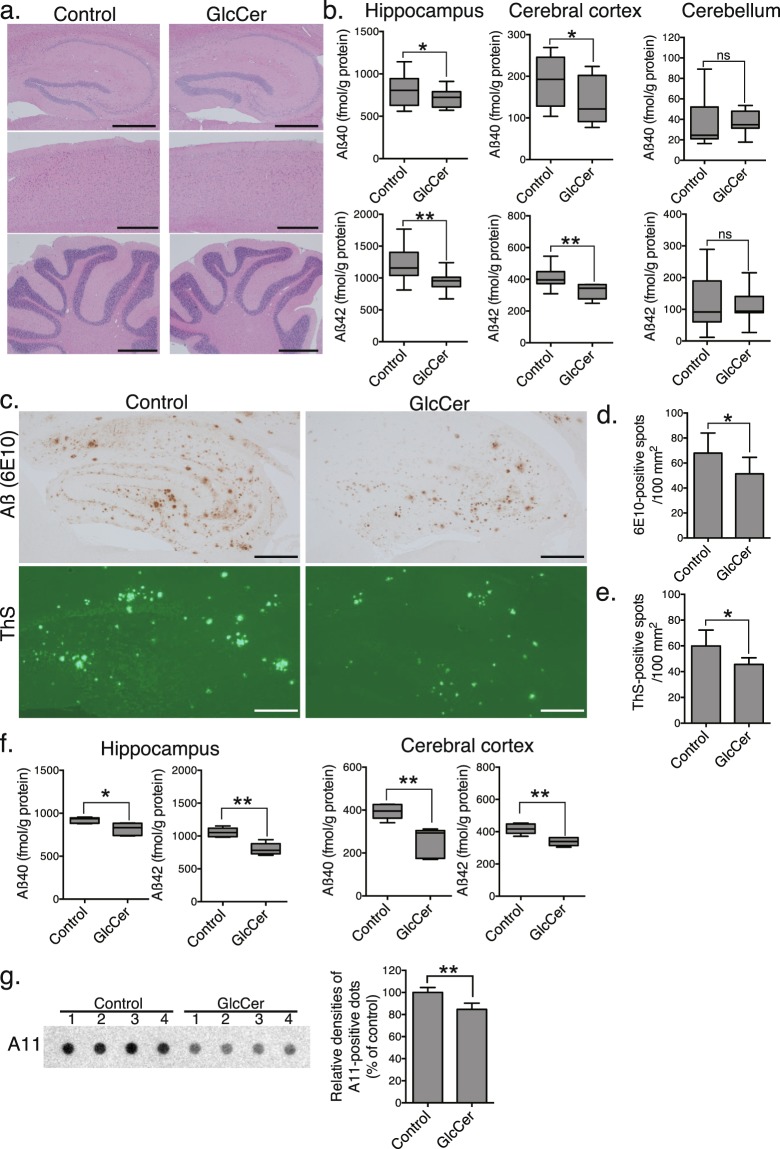
Oral administration of plant GlcCer reduces Aβ accumulation in APP mice. (**a**) Haematoxylin and eosin staining of the brains of APP mice orally administered GlcCer or vehicle (control) for 14 days. Scale bars, 500 µm. (**b**) The levels of Aβ40 and Aβ42 in brain tissues were measured by ELISA (*n* = 10 animals, 6 males and 4 females, each group). (**c**) Representative images of Aβ-immunostained and thioflavin-S (ThS)-stained hippocampal sections. Bars, 200 µm. Quantification of Aβ-immunopositive (**d**) and ThS-positive (**e**) areas in hippocampi (*n* = 10 animals, 6 males and 4 females, two sections per brain). (**f**) Concentrations of Aβ in hippocampi and cerebral cortices of mice administered purified GlcCer or vehicle were measured by ELISA (*n* = 6 animals, 4 males and 2 females, each group). (**g**) Dot blot analysis using anti-oligomer (A11) antibody of hippocampal lysates of GlcCer or vehicle-administered mice. Right graph exhibited quantification of A-11 positive staining in dot blots. (*n* = 4 animals, 2 males and females, each group). Data are presented as means ± SDs. ns, not significant; *P < 0.05; **P < 0.01 by *t* tests.

To investigate the effects of plant ceramides *in vivo*, APP^SweInd^ transgenic mice were orally administered 11% (wt/wt) GlcCer-containing *A. konjac* extracts (KE) (1 mg GlcCer/day) for 14 days. Treatment did not alter body weights (see Supplementary Fig. S2) or the morphology of the hippocampus, cerebral cortex and cerebellum as assessed by haematoxylin and eosin staining (Fig. 3a). In addition, we performed a modified SHIRPA testing that covers about 20 measures of sensorimotor functions and reflexes (Supplementary Table S1). Most of the behaviour aspects in GlcCer-treated mice were similar as the control, indicating administration of GlcCer did not result in general neurological defects in the mice. Next, the levels of Aß in hippocampus, cerebral cortex and cerebellum tissues were measured by ELISA. The levels of Aβ40 and Aβ42 were substantially decreased in the hippocampi and cerebral cortices of the KE-treated mice compared with those in the controls as measured by ELISA (Fig. 3b). Histochemical observations revealed that the hippocampi of KE-treated mice also had markedly decreased Aβ immunoreactivity and a markedly reduced density of thioflavin-S-positive plaques (Fig. 3c–e). Notably, oral administration of purified GlcCer resulted in similar declines in Aβ levels in the hippocampi and cerebral cortices of APP mice (Fig. 3f). Dot blot analysis using an oligomer-specific antibody A11, revealed that as well as Aß fibrils, the level of oligomeric Aß was also decreased in the GlcCer-administered mouse brains (Fig. 3g). As accumulated lines of evidence demonstrate that neurodegeneration and synaptic impairment in AD pathogenesis are directly caused by soluble Aß oligomers^25,26^, the above finding indicated that orally administered plant GlcCer attenuates accumulation of Aß-related pathological species in the APP overexpressing mice.

### Dietary plant GlcCer reduces brain inflammation, synaptic toxicity and cognitive impairment in APP mice

**Figure 4:**
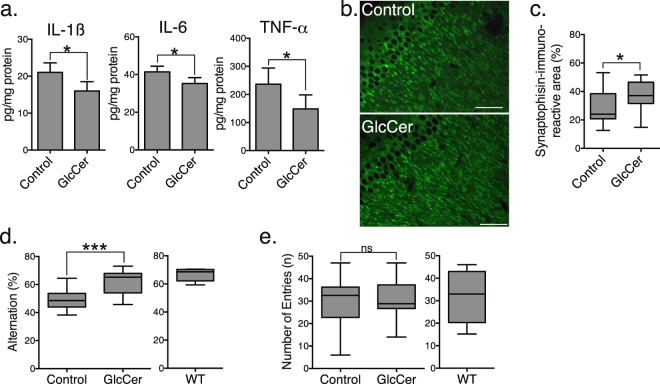
Plant GlcCer attenuates inflammation and cognitive impairment in APP mice. (**a**) Levels of proinflammatory cytokines in the cerebral cortices of APP mice administered purified GlcCer or vehicle were measured by ELISA (*n* = 10 animals, 6 males and 4 females, each group). (**b**) Representative hippocampal sections of GlcCer- or vehicle-treated APP mice stained with antibody against synaptophysin. Scale bars, 100 µm. (**c**) Densities of synaptophysin-positive presynaptic terminals in the hippocampal sections (two sections/mouse, five mice, 3 males and 2 females, per group). Spontaneous alternation (**d**) and numbers of arm entries (**e**) of GlcCer- and vehicle-treated APP mice (*n* = 15 animals, 8 males and 7 females, each) and age-matched wild-type mice (*n* = 5, all males) in the Y-maze to evaluate spatial memory. The data represent the means ± SDs. ns, not significant; *P < 0.05 by *t* tests.

APP mice exhibit deficits in spatial memory along with neuronal inflammation and synaptic loss in brain tissue resulting from the high expression of Aβ^27–29^. Thus, we performed ELISAs to assess neuroinflammation in the cerebral cortices of our treated and untreated APP mice. The levels of interleukin (IL)-1β, IL-6 and tumour necrosis factor (TNF)-α were substantially lower in the cortices of mice orally administered purified GlcCer than in the vehicle-treated control mice (Fig. 4a). GlcCer-treated APP mice also exhibited higher synaptic densities in the hippocampus, as assessed by immunostaining for the synaptic marker protein synaptophysin, suggesting that there was recovery from synaptic impairment (Fig. 4b,c). Next, we analysed whether GlcCer improve Y-maze performance of APP overexpressing mice. Consistent with the synaptic recovery, the performance of GlcCer-treated APP mice in the tests of cognitive function was improved. Whereas APP mice showed lower rates of spontaneous alternation than age-matched control mice in the Y-maze test, an indication of impaired short-term spatial memory, GlcCer-treated APP mice had markedly higher rates than the vehicle-treated controls (Fig. 4d), demonstrating the effect of GlcCer on the recovery of cognitive functions in the APP overexpressing mice. There was no difference between the groups in the number of entries into each of the arms (Fig. 4e).

### Dietary plant GlcCer modulates levels of EVs in mouse serum and brains

**Figure 5:**
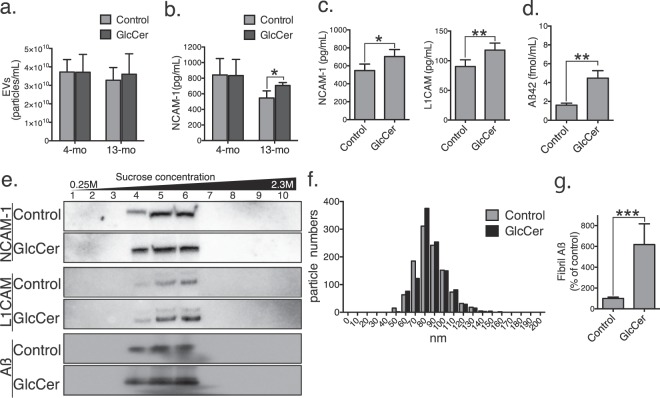
Plant GlcCer alters levels of EVs in serum and brain. Numbers of EVs (**a**) and exosomal expression of NCAM-1 (**b**) from serum samples of control or GlcCer-treated wild-type mice (n = 5, all males, each group). (**c**,**d**) NCAM-1, L1CAM, and Aβ42 levels in serum EVs of 13-month old APP mice, which were administrated GlcCer or vehicle (n = 5, 3 males and 2 females, each group). (**e**) NCAM-1, L1CAM, and Aβ were detected in sucrose gradient ultracentrifugation fractions of GlcCer- and vehicle-treated APP mouse brains. (**f**) Histogram of the size of EVs in fractions 3, 4 and 5 from panel (e). (**g**) Exosome-associated Aß fibrils in fractions 3, 4 and 5 from panel e were measured by PS-capture exosome ELISA and amyloid fibril-specific antibody (n = 5, 3 males and 2 females, each group). Data are the means ± SDs. *P < 0.05; **P < 0.01, ***P < 0.001 by *t* tests.

To confirm that the observed reduction in Aβ levels was thus due to enhanced clearance by EVs, we collected serum from 4- and 13-month-old mice 5 h after the second dose of purified GlcCer (oral, 1 mg every 24 h). Although the amounts of the EVs were similar between GlcCer- and vehicle-treated mice (Fig. 5a), the expression of neural cell adhesion molecule 1 (NCAM-1), a neuronal marker, was increased in the serum EVs from 13-month-old GlcCer-treated wild type mice (Fig. 5b). As well as NCAM1, the expression of another neuronal marker L1 cell adhesion molecule (L1CAM) was also increased in the EVs isolated from the GlcCer-administered APP overexpressing mouse serum (Fig. 5c). The level of serum Aß was higher in the GlcCer-administered APP overexpressing mice than vehicle-treated mice (Fig. 5d).

We next isolated EVs from the brain tissues of the GlcCer- and vehicle-administered APP mice by sequential centrifugation and filtration (described in Materials and Methods). Western blot analyses revealed that the expression levels of NCAM-1, L1CAM, and Aβ were substantially increased in the brain EVs from GlcCer-treated mice (Fig. 5e, Supplementary Fig. S3). The particle sizes of the isolated EVs ranged from 60 to 130 nm in diameter (Fig. 5f), similar to those observed *in vitro*. In addition, there was a remarkable increase in the plant ceramide-treated exosomes that are associated with amyloid fibrils as determined by ELISA using Tim4, a receptor for exosomal lipid phosphatidylserine, and an antibody against amyloid fibril (Fig. 5g). These findings suggest that dietary plant GlcCer increases neuronal EVs harbouring Aβ for clearance *in vivo*.

## Discussion

The results from the present study reveal a novel physiological function of plant ceramides, namely, that they decrease brain Aβ levels to prevent AD-like pathologies. Specifically, we showed that ceramides extracted from *Amorphophallus konjac* increase neuronal EVs that bind to Aβ, resulting in lower extracellular Aβ levels in a neuronal and glial coculture system as well as reduced amyloid deposition and Aβ-related pathologies in the brains of APP mice. Dietary plant GlcCer also increased the expression of NCAM-1 and synaptophysin in the brains of these mice as well as the amounts of Aβ in the EVs isolated from serum and brain tissues. These findings suggest that dietary plant ceramides suppress Aβ pathology by promoting production of EVs.

We previously reported that sphingolipid metabolism, particularly, the elevation of ceramides, modulates release of EVs from neuronal cells^8^. Two of the enzymes involved in this metabolism, namely, neutral sphingomyelinase-2 and SMS2, are expressed in plasma and endocytic membranes in neurons and have opposite functions for the conversion of sphingomyelin to ceramide^30,31^. The role in the production of EVs is greater for SMS2 than for isoform SMS1, which is dominantly expressed in the Golgi apparatus^8,32^. Exosomes are produced initially by intraluminal budding of the endosomal membrane. The hydrolysis of sphingomyelin and the accumulation of ceramide in endosomes initiate the budding of the endosomal membranes^11^, and elevated levels of ceramides at particular subcellular regions, including plasma membranes and endocytic compartments, are thought to be involved in exosome generation. The tracking of fluorescence-labelled lipids revealed that exogenously added ceramides, especially those containing long fatty acids, are taken up into cells and trafficked via the endocytic pathway to lysosomes^33^. Thus, exogenously applied plant ceramides might similarly be internalised by cells and generate exosomes at endosomal membrane regions. However, the contribution of ceramide-derived metabolites that might mediate exosome production should also be considered, such as sphingosine 1-phosphate^34,35^, a minor metabolite generated from ceramide by sequential degradation and phosphorylation. Sphingosine 1-phosphate activates a G_i_-coupled receptor to induce intraluminal budding of endosomal membranes resulting in multivesicular bodies. Whether plant ceramides are metabolised identically to endogenous ones in mammalian cells needs to be studied.

Parkinson disease (PD)-related protein α-synuclein, is also known to associated with neuronal exosomes^36^. The exosomes can induce aggregation of α-synuclein as well as Aß, and also promote extracellular efflux of α-synuclein^37–39^. A challenging study in the future will be determined whether ceramide-dependent exosome release is involved in assembly and clearance of alpha-synuclein in PD pathogenesis. On the other hand, mutations in glucocerebrosidase, a GlcCer-degrading enzyme, are known to be the risk factors for PD. It has been reported that metabolism of plasma ceramide and monohexosylceramide, GlcCer and galactosylceramide, is altered in sporadic PD^40^. Orally administerd GlcCer are digested in digestive tract and absorbed into body as its components, glucose, fatty acid, and sphingoid bases. However, the possibility that plant GlcCer might affect metabolism of endogenous sphingolipids including GlcCer would need to be carefully examined.

Plant sphingolipids might affect endogenous *de novo* ceramide synthesis in mammalian cells. Sphingoid bases, such as 4,8-sphingadienine in *Amorphophallus konjac*, accelerate endogenous ceramide synthesis in cultured keratinocytes by upregulating ceramide synthesis-related genes, including ceramide synthase^41–43^. Treatment of human epidermal keratinocytes or a 3-dimensional skin culture model with 4,8-sphingadienine also elongates very-long-chain fatty acid protein and consequently results in the accumulation of d18:1 sphingolipids (such as ceramides and GlcCer)^41^. Dietary plant GlcCer increases endogenous ceramides in the stratum corneum in mice and human subjects^15,44^. Further investigation is required to clarify which species of plant sphingolipid metabolites are taken up by neurons to promote production of EVs. In addition, Sphingolipids including ceramides display structural diversity depending on their sources. Mammals mainly produce sphingosine (d18:1), sphinganine (d18:0), and phytosphingosine (t18:0)^45^. Plants, fungi, and marine organisms produce structurally distinct sphingoid bases such as sphingadienine (d18:2), 9-methyl sphingadienine (9Me-d18:2), sphingatrienine (d18:3), and 9-methyl sphingatrienine (9Me-d18:3)23). To examine the differences of their effects on exosome release and AD pathologies would be a future challenge.

Plant GlcCer was included in daily food sources and available as an oral supplement for skin barrier improvement. Mice with atopic dermatitis orally administered 4 mg plant GlcCer daily for 2 weeks showed reduced TWEL and increased stratum corneum flexibility^20^. Notably, the dose applied to the APP mice in the present study was much lower (1 mg/day, 2 weeks). A comparable dose in humans to prevent Aβ deposition is estimated to be more than 0 mg per day. It is estimated that food sources provide as little as 50 mg plant sphingolipids daily^46^. The data presented here suggest that additional intake of plant sphingolipids from foods or sphingolipid-based dietary supplements may be useful for the prevention of dementia. Further careful studies are needed to investigate the dosage that is optimal to reduce Aß and the side effects, however, plant ceramides might be a candidate functional food material to prevent AD-like pathology.

## Materials and Methods

### Animals and treatments

Hemizygous transgenic mice expressing human APP harbouring the Swedish and Indiana (KM670/671NL, V717F) mutations (APP^SweInd^) labeled as strain B6.Cg-Tg (PDGFB-APP^SwInd^) 20Lms/2 J (MMRRC Stock No: 34836-JAX) were from Jackson Laboratory (Bar Harbor, ME), and C57BL/6 mice were purchased from Japan SLC (Hamamatsu, Japan). All animals were maintained in barrier facilities, housed in a room kept at 23 ± 1 °C with a 12- h light/dark cycle and allowed free access to tap water and food (AIN-93M). Animal protocols were approved by the animal care committees of Hokkaido University and all experiments were performed in accordance with guidelines and regulations of the animal care committees of Hokkaido University.

APP mice (13 months) were isolated one per a cage and received a daily oral dose of 9.2 mg KE containing 11% (vol/vol) GlcCer or 1 mg purified GlcCer in 150 μl 1% tragacanth gum/water (or vehicle only for control animals) via an intragastric feeding needle for 14 days. Before feedings, we mixed and dispersed KE and purified GlcCer in 1% tragacanth gum/water by vortex and sonication. To analyse EVs in sera and brain tissues, blood and brain samples were collected from C57BL/6 (4 or 13 months) or APP (13 months) mice 5 h after the second daily oral dose of purified GlcCer.

### Cell cultures

Neuroblastoma SH-SY5Y cells with and without stable transfection of human APP770 were maintained in Eagle’s minimum essential medium/Ham’s F-12 medium (Thermo Scientific, Waltham, MA) supplemented with 1% nonessential amino acids and 10% fetal bovine serum. The murine microglial BV-2 cell line was purchased from Istituto Nazionale per la Ricerca sul Cancro (Genova, Italy) and cultured in RPMI 1640 (Thermo Scientific) supplemented with 10% foetal bovine serum and l-glutamine. Primary neuronal cultures were prepared from cerebral cortices of embryonic day 15 mice using a dissociation solution (Sumitomo Bakelite, Tokyo, Japan). The cells (5 × 10^5^/cm^2^) were plated on polyethyleneimine-coated dishes and cultured for 7 days in neurobasal medium with 25 mM KCl, 2 mM glutamine and B27 supplement (Thermo Scientific). For Transwell cultures, APP-expressing SH-SY5Y cells (5 × 10^5^/cm^2^) cultured on 24-well plate inserts (0.5 µm pore; Corning, NY) and BV-2 cells (1 × 10^6^) placed below the inserts were treated for 24 h with 10 µM GlcCer or ceramides in Eagle’s minimum essential medium/Ham’s F-12 medium.

### Preparation of plant ceramide for *in vitro* assay

Plant ceramides were prepared in our laboratory according to a published procedure^18^. Briefly, *A. konjac*-derived GlcCer (Nagara Science, Gifu, Japan) was degraded with EGCase I isolated from a mutant *Rhodococcus erythropolis* L-88 strain containing pTip-EGCaseI plasmid^47^. The samples were then extracted by the Bligh–Dyer method, subjected to medium-pressure liquid chromatography on a Hi-Flash S column (Yamazen Corp., Osaka, Japan) and separated into two fractions containing ceramides and GlcCer. The pooled fractions containing ceramides were dried and used for culture cell assays.

### Purification of GlcCer from KE for *in vivo* assay

KE containing 11% GlcCer was obtained from Daicel Corporation (Osaka, Japan). The extract was diluted in a test tube using 10 volumes of cold acetone before centrifugation. The supernatant was discarded, and polar lipids were collected. Glycerolipids were hydrolysed by incubating at 37 °C for 2 h with 0.4 M KOH in methanol. The solvent was evaporated, and the residue was purified by silica gel column chromatography using a chloroform/methanol/acetic acid solvent gradient (190:9:1 → 9:1:0 → 7:3:0 → 1:1:0 [vol/vol]) as an eluent. The fractions eluted with the 9:1:0 and 7:3:0 solutions were mixed and evaporated. Methanol (95%) was then added for further purification by HPLC. The obtained GlcCer was evaporated and used for *in vivo* tests.

### Isolation of EVs

EVs were collected by differential ultracentrifugation^48^ from supernatants of SH-SY5Y cells treated with sphingolipids in serum-free medium for 24 h. The supernatants were sequentially centrifuged at 2,000 × *g* for 10 min and 10,000 × *g* for 30 min at 4 °C to remove cells and debris and then again at 100,000 × *g* for 1 h at 4 °C to pellet the EVs.

EVs-rich fractions were prepared from mouse brain as previously described^49^. The left hemispheres were dissociated with 20 U/ml papain (Sigma-Aldrich, St. Louis, MO) at 37 °C for 15 min and then filtered through 40 µm and 0.2 µm mesh filters. The crude EVs were then isolated by differential centrifugation as described above and subsequently purified by sucrose density gradient (0.25–2.3 M sucrose in 20 mM HEPES, 10 ml). After centrifugation, 1 ml fractions were collected, diluted with PBS and precipitated by centrifugation for 1 h at 100,000 × *g*.

Pellets were resuspended in appropriate buffers for biochemical analyses. The particle sizes and densities of the isolated EVs suspended in PBS were measured with a qNano system (Izon Science, Cambridge, MA) using NP200 nanopores and qNano Izon analysis software.

### SDS-PAGE and Western blot analysis

SDS-PAGE and Western blot analysis were performed according to the standard methods of Laemmli. Target proteins were detected using antibodies against Alix (BD Bioscience, San Jose, CA), N-CAM1(Santa cruz, California, CA), and L1CAM (2C2, abcam, Cambridge, UK). Horseradish peroxidase-conjugated cholera toxin B subunit (Sigma-Aldrich) was used to detect GM1. Bands were visualised using Chemi-Lumi One (nacalai tesque, Kyoto, Japan) and an LAS4000 imaging system (Fuji Film, Tokyo, Japan).

### Dot blot analysis

The hippocampal lysates (20 µg protein) were dotted onto a nitrocellulose membrane, and the membrane was incubated with primary antibodies against Aß oligomer (A11) and then with HRP-conjugated secondary antibodies. Chemiluminescence was detected and analysed using a combination of an Chemi-Lumi One and a LAS4000 imaging system.

### GSL-glycan purification and MALDI-TOF MS analysis

The harvested culture cells and the EVs were suspended in acetate buffer (50 mM, pH 5.5) containing 0.2% Triton X-100 and sonicated with an Ultrasonic Homogenizer (TAITEC, Saitama, Japan). To release GSL-glycans, 5 μL of endoglycoceramidase I (EGCase I) from *Rhodococcus equi* was added to their lysates of cultured cells and their EVs corresponding to 40 μg protein^47^. EGCase digestion was performed at 37 °C for 16 h. After deglycosylation, ethanol was added to the reaction mixture and incubated at −30 °C for 4 h. For recovery of GSL-glycans, the supernatant fractions were separated by centrifugation and dried with a centrifugal evaporator. The concentrated supernatant containing GSL-glycans were resuspended in 50 μL of H_2_O and subjected to a glycoblotting procedure as previously described^50^. The analysis of GSL-glycans was performed by MALDI-TOF MS using an Ultraflex II TOF/TOF mass spectrometer, which was controlled by the FlexControl 3.0 software package (Bruker Daltonics, Bremen, Germany). All spectra were obtained as positive ions and masses were annotated using the FlexAnalysis 3.0 software package (Bruker Daltonics). The SphinGOMAP (http://www.sphingomap.org/) online databases were used for structural identification of GSL glycans^51^.

### Seed-free Aβ preparation

Seed-free Aβ solutions were prepared according to a published reports^52,53^. Briefly, synthetic Aβ_1-40_ (Peptide Institute, Osaka, Japan) was dissolved in an ice-cold 0.02% ammonia solution to a final concentration of 500 µM. To remove undissolved Aβ aggregates, the solution was centrifuged at 540,000 × *g* for 3 h, and the supernatants were stored at −80 °C until use. Immediately before use, the aliquots were thawed and diluted with culture medium or Tris-buffered saline (TBS) (150 mM NaCl and 10 mM Tris-HCl, pH 7.4).

### Immunoelectron microscopy

EVs from ceramide-treated SH-SY5Y cells incubated with Aβ for 1 h at 37 °C were stained with phosphotungstic acid. The EVs were incubated with anti-Aβ antibody (4G8) and then 10 nm gold-coupled anti-IgG. Images were captured with a JEM-ARM200F (JEOL Ltd., Tokyo, Japan) transmission electron microscope.

### Thioflavin T assay

Seed-free Aβ solutions (25 µM) were incubated at 37 °C in 100 µl TBS containing 0 or 10 µl EVs solutions collected from culture supernatants of 1 × 10^7^ cells, and the fluorescence intensities of thioflavin T were measured using an Appliskan spectrofluorophotometer (Thermo Scientific). The optimum fluorescence intensities of amyloid fibrils were measured at excitation and emission wavelengths of 446 and 490 nm, respectively.

### ELISAs

The levels of Aβ in Transwell culture medium or tissue supernatants were determined with a sandwich ELISA (Wako, Osaka Japan). For tissues, hippocampi or cortices were homogenised in 4 M guanidine-HCl buffer (pH 8.0) with an ultrasonic homogeniser (Taitec, Saitama, Japan) and incubated at room temperature for 3 h. The homogenates were diluted with 0.1% bovine serum albumin in PBS and centrifuged at 16,000 × *g* for 20 min. The resulting supernatants were then applied to the ELISA to measure Aβ levels or to Multi-Analyte ELISArray (Qiagen, Hilden, Germany) to measure levels of the proinflammatory cytokines TNF-α, IL-6 and IL-1β according to the manufacturer’s instructions.

EV-associated NCAM-1 and L1CAM were measured by ELISA from MyBiosource (San Diego, CA) and LifeSpan BioSciences (Seatle, WA). Total exosome levels were analysed using PS Capture Exosome ELISA Kit with anti-CD9 antibody (Wako) according to the manufacture’s instruction. For detection of exosomal amyloid fibrils, conformational specific anti-amyloid fibril antibody (mOC87, abcam) and HRP-conjugated anti-rabbit IgG were used as detection antibodies in PS Capture Exosome ELISA Kit.

### Tissue staining

The left hemispheres of mouse brains were fixed in 4% paraformaldehyde in 0.1 M PBS, dehydrated and embedded in paraffin for sectioning and staining with haematoxylin and eosin. Serial 10 µm-thick sections were immunostained with monoclonal antibody against Aβ (6E10; Covance, Princeton, NJ) after a brief formic acid treatment, and the signals were visualised using an ABC elite kit (Vector Laboratories, Burlingame, CA). Sections were also stained with 0.015% thioflavin-S in 50% ethanol for 10 min and developed with two 4 min washes in 50% ethanol. Aβ-immunopositive and thioflavin-S-positive plaques from fluorescence images were quantified as the percentage of the positive area (positive pixels) relative to the examined area (total pixels) using ImageJ software.

### Evaluation of synaptic densities

Densities of synaptophysin-immunopositive synapses were quantified according to the methods of Mucke *et al*.^54^ with minor modifications. Brain tissue sections were immunostained with a monoclonal antibody to synaptophysin (D35E4; Cell Signaling Technology, Danvers, MA) and incubated with Alexa Fluor 488-bound anti-IgG. For each mouse, nine confocal images were captured from three sections, and each image covered 5,500 µm^2^ of the molecular layer of the dentate gyrus. Synaptic densities were estimated as a percentage of the immunostained area as described above.

### Y-maze spontaneous alternation test

Short-term spatial memory performance was evaluated by recording spontaneous alternation behaviour in a Y-maze (PanLab, Barcelona, Spain) comprising black painted plastic arms (35 × 10 × 11 cm [l × w × h]). Each mouse, naive to the maze, was placed in one arm, and the sequence and number of arm entries were counted during an 8 min session. The alternation rate was calculated as the ratio of actual to possible alternations (defined as the total number of arm entries minus two)^55^.

### Modified SHIRPA

We performed the modified SHIRPA testing^56^. Briefly, after two weeks of GlcCer or vehicle treatment, mice were individually placed in a clear cylindrical viewing jar, to observe the following features: coat appearance, white belly, hair length, tremor, body position, whisker appearance, palpebral closure, lacrimation. Afterwards, mice were placed in an arena from a height if approximately 25 cm and the following aspects were observed: transfer arousal, locomotor activity, gait, grasp reflex, biting, tail elevation and touch escape. Finally, the following features were also recorded: skin color, trunk curl, limp grasping, pinna reflex, corneal reflex, and vocalization.

## Supplementary information

Supplementary information

## Data Availability

All data generated or analysed during this study are included in this published article (and its Supplementary Information Files).
