# Supplementary material for: Plant sphingolipids promote extracellular vesicle release and alleviate amyloid-β pathologies in a mouse model of Alzheimer’s disease
Source: Sci Rep. 2019 Nov 14;9:16827. doi: 10.1038/s41598-019-53394-w (PMC6856149; doi:10.1038/s41598-019-53394-w)
Supplement: Supplementary file 1 — Supplementary information [file 41598_2019_53394_MOESM1_ESM.pdf]

Supplementary information

Plant sphingolipids promote extracellular vesicle release and alleviate amyloid-ß pathologies in a mouse model of Alzheimer's disease

Kohei Yuyama, Kaori Takahashi, Seigo Usuki, Daisuke Mikami, Hui Sun, Hisatoshi Hanamatsu, Junichi Furukawa, Katsuyuki Mukai, Yasuyuki Igarashi

Supplementary Figures and Table:

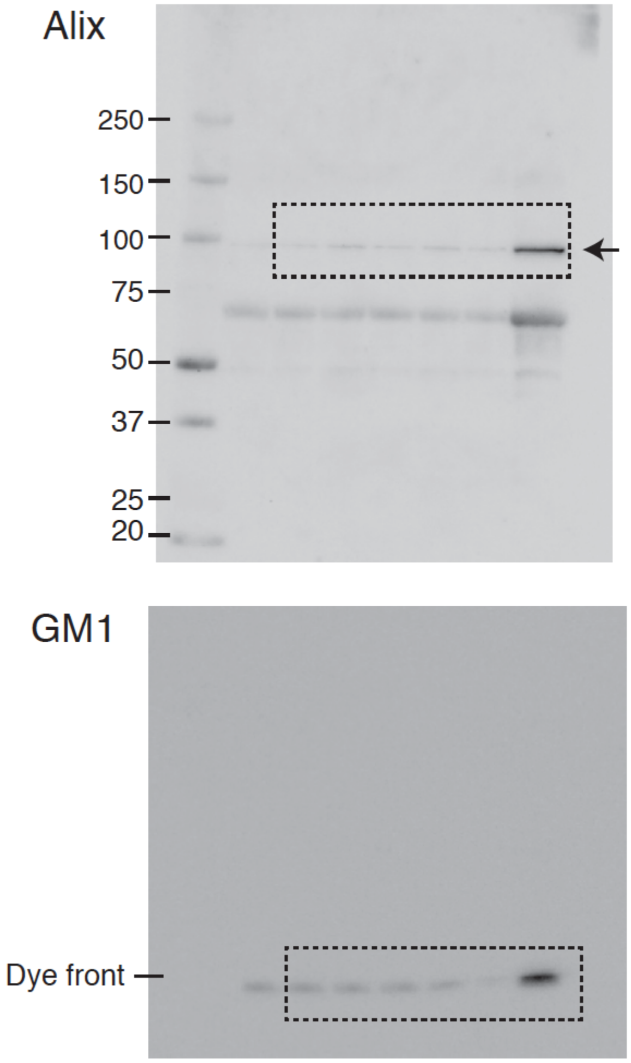

Supplementary Fig.S1

Full-length blots for Fig. 1d.

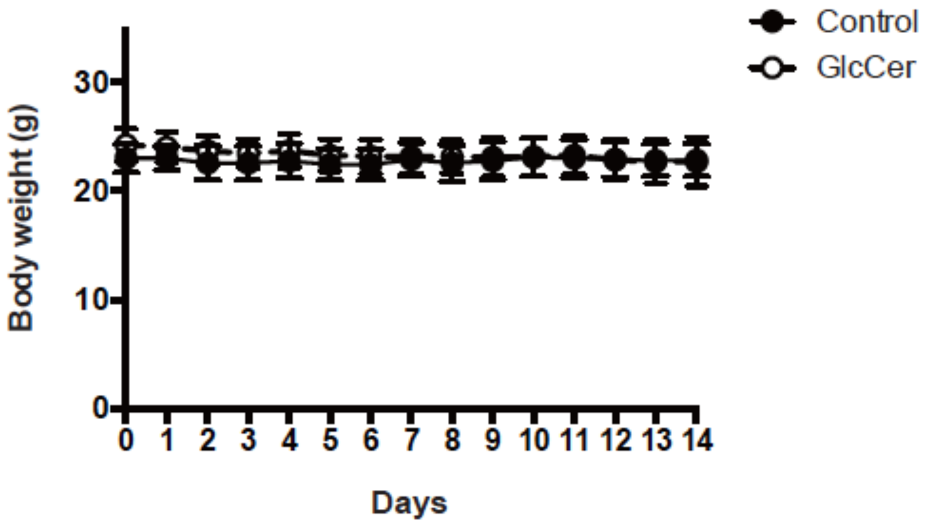

Supplementary Fig.S2

Body weights during plant GlcCer administration.

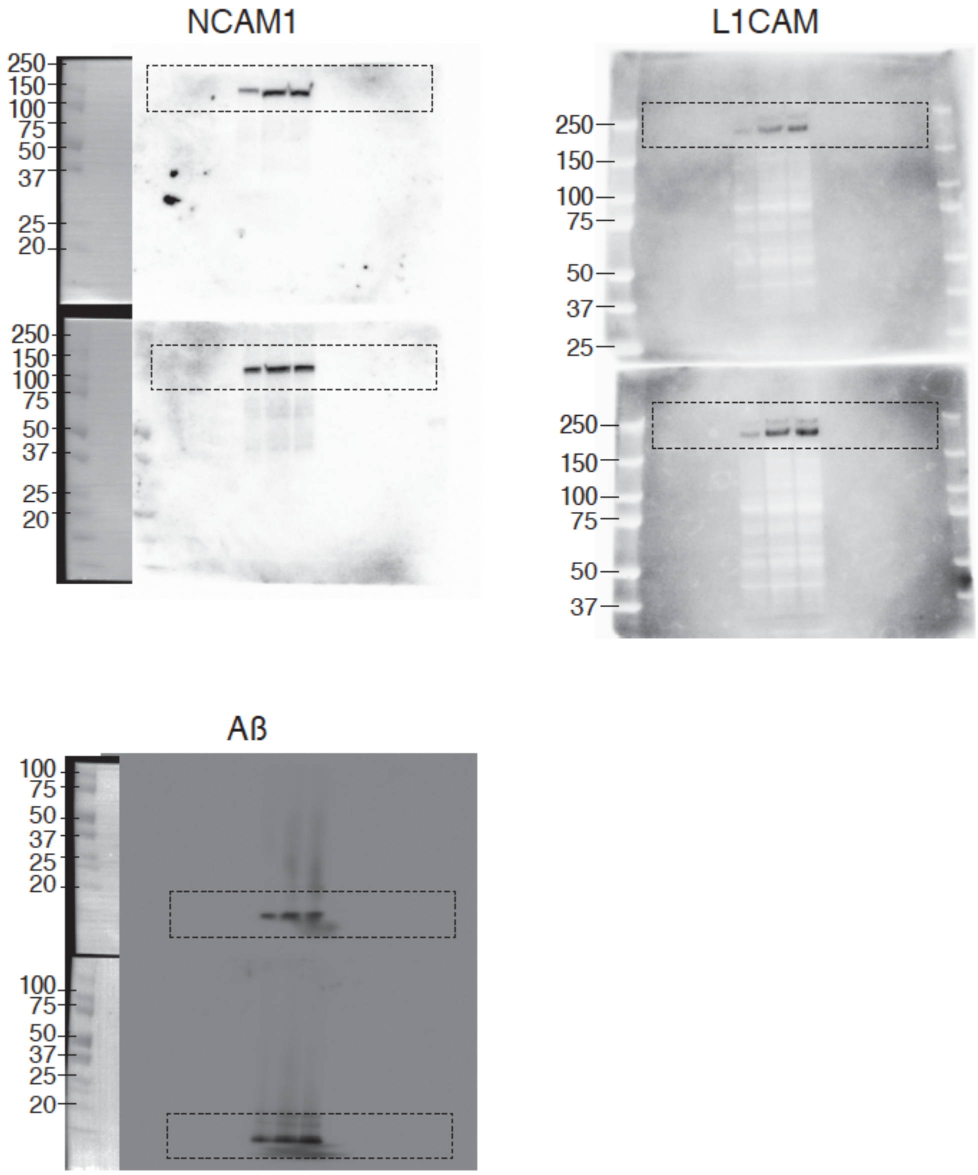

Supplementary Fig.S3

Full-length blots for Fig. 5e.

Modified SHRPA results

SHIRPA			Control	GlcCer
In the Viewing Jar	Coat color	Abnormal	0/14	0/14
	White belly	No	14/14	14/14
	Hair length	Abnormal	0/14	0/14
	Tremor	Present	0/14	0/14
	Body position	Flat	14/14	14/14
	Whiskers	Abnormal	0/14	0/14
	Palpebral closure	Open	14/14	14/14
	Lacrimation	Present	0/14	0/14
In the Arena	Transfer arousal	< 1 sec	4/14	3/14
		1-5 sec	10/14	11/14
		>5 sec	0/14	0/14
	*Locomotor activity	sec	16.49	15.96
	Gait	Fluid movement	14/14	14/14
	Grasp reflex	Present	14/14	14/14
	Biting	Present	3/14	2/14
	Tail elevation	Elevated	10/14	9/14
		Extended	3/14	3/14
		Drag	1/14	2/14
	Touch escape	Mild	2/14	1/14
		Moderate	12/14	13/14
Above the Arena	Skin color	Pink	14/14	14/14
	Trunk curl	Present	0/14	0/14
	Limb grasping	Present	0/14	0/14
	Pinna reflex	Present	14/14	14/14
	Cornial reflex	Present	14/14	14/14
	Vocalization	Present	3/14	2/14

*Unpaired t-test: p= 0.8036 (Control n=14, GlcCer n=14)

Supplementary Table S1
